# Nasal Foam Packing for Epistaxis: What are the Ideal Characteristics?

**DOI:** 10.1055/s-0045-1809999

**Published:** 2025-09-10

**Authors:** Flavio Serafini, Isabele Campos Araújo, Flavia Minhoto, Ana Luiza Figueira Santos

**Affiliations:** 1 Universidade de Taubaté (UNITAU), Taubaté, SP, Brazil

**Keywords:** tampons, epistaxis, hypertension

## Abstract

**Introduction:**

Epistaxis is a common otorhinolaryngological emergency, usually caused by digital trauma in children, and hypertension in the elderly. General practitioners in emergency rooms assist most of the cases without specific management.

**Objectives:**

Describe suitable properties for efficient nasal packing in adult epistaxis.

**Methods:**

Two cadaver heads, preserved by freezing process, were thawed for this research. When the room temperature was reached, the nasal cavities were evaluated by a nasal endoscope, which showed a length of 4.0 × 11.0 cm for the male and 4.0 × 8.5 cm for the female head. Through an orifice made in the left maxillary bone to access the maxillary sinus, a blue-dyed saline solution was applied using a sphygmomanometer (Premium) to control and mimic blood pressure. The nasal cavity was first packed with two foams with different thicknesses and densities evolved by a condom, then packed by the Merocel packing (Medtronicent Surgical Products Inc.). The pressure was administered and gradually increased until a saline leak was observed in the oropharynx. The best way to insert tampons was also evaluated to avoid extensive trauma.

**Results:**

Despite the difficulty of the application being similar in both nasal packings made with the condom and foam, t33-densityity foam, 2.0 cm thick and 11.0 cm lon resisted to a higher pressure (250mmHg) than the commercial packing Merocel (220mmHg).

**Conclusion:**

The most suitable foam for packaging wrapped with condoms is 2.0 cm thick, as it supported the blood pressure commonly found in epistaxis.

## Introduction

Epistaxis, considered the main otorhinolaryngological emergency, is defined as bleeding that originates in the nasal mucosa, which covers the vascularization system of the nose. It may occur due to hemostasis alterations, whether due to loss of vascular status, nasal mucosa abnormalities, or even changes in coagulation factors. It is classified as anterior and posterior based on both symptoms and the anatomical characteristics of nasal vascularization. Anterior epistaxis is the most common occurrence due to the high vascularity and vulnerability, and it may consist of causes such as mucosal dryness and digital trauma. 1 2

The mucosa that covers the nasal cavities is part of a complex irrigation network, originated by the systems of the internal and external carotid arteries which, with multiple anastomoses, form the erectile tissue of the superior, the middle, and the inferior turbinates (located on the lateral wall), and the nasal septum. The sphenopalatine artery (external carotid artery branch), the anterior and posterior ethmoid arteries (external carotid artery branch), and the superior labial artery (facial artery branch) are mainly responsible for nasal cavities blood supply. The greater palatine, the sphenopalatine, and the superior labial arteries constitute Kiesselbach's plexus (or Little's area) through an anastomotic network in the anterior nasal septum, a region which is responsible for most of the anterior bleedings, whose vessels are lined with a thin mucous membrane; whereas most of the posterior bleedings are originated in the Woodruff's plexus, located next to the choana in the posterior region. 3

Mostly, nasal bleeding is easily controlled, sometimes not requiring medical assistance. However, 6% of the cases require an intervention from a specialist to contain the bleeding; and only 1% need hospital admission, with a mortality rate of less than 0.01%. 4 Hospital admission cases usually include severe epistaxis, which is difficult to control and is associated with some predisposing factors. Among them are Systemic Arterial Hypertension (SAH), coagulopathies, nasal trauma, and previous surgeries. A blood transfusion may be necessary in these cases, which shows the high morbidity related to bleeding, and therefore challenging to manage. 4

Normally epistaxis occurs at any age without gender predilection, varying only in incidence and cause. However, a high frequency is observed in children, with no severity, and in patients older than 50 years, cases which should receive more attention. For the younger group, non-traumatic epistaxis is common in both sexes, especially in groups with more precarious socioeconomic conditions. In addition, bleeding is more common when the nasal mucosa becomes fragile, a fact favored by the driest months of the year.

Epistaxis can also occur due to local factors, such as digital trauma, nasal septum deviation, neoplasms, inflammation, rhinosinusitis, foreign bodies, polyps, chemical irritants (as of cigarettes, cocaine), as well as systemic factors such as coagulopathies, hemophilia, liver diseases, renal failure, alcoholism, and vascular abnormalities. 1 5 It is also the most common symptom in approximately 60% of patients who suffer from Von Willebrand. 6 Yet 80-90% of the cases are identified as idiopathic, followed by causes such as trauma or arterial hypertension. 7

Bleeding control is often related to the cause and its anatomical location, followed by electrical cauterization. Silver nitrates can be used as a first-line treatment for minor bleeding. In case bleeding local is not found, the packing can be done with gauze soaked in Vaseline, nitrofurazone, or antibiotic ointment, completely filling the nasal cavity. Other alternatives are packings made with glove fingers filled with gauze, a condom-coated sponge for domestic use, and a Merocel© packing (polyvinyl hydroxylate acetate). 8 In addition, in larger nostrils, packing with a nasal balloon catheter with fibrin colloidal material and a double system of cuffs for anterior and posterior packing can be used, inflated with air, forming a hemostatic dressing, in a less traumatic but less effective way, as they deflate with the time. 9 In severe epistaxis unresponsive to conservative treatment, a sphenopalatine artery endonasal ligation may be performed, as it does not present post complications such as rebleeding, edema, facial anesthesia, oroantral fistula. 4 In some cases, emergency management is taken to contain bleeding in non-localized bleeding or cause, leading to an empirical approach to compressive and non-surgical packing of the nasal cavity. This packing can bring pain, hypoxia, toxemia, and even alar necrosis. 10

Due to the absence of a protocol aimed at nasal bleeding in 85% of emergency care units, the emergency physician is usually responsible for the tampons placement, which does not provide the best cost-benefit ratio for both the patient and the unit care, due to lack of knowledge of ideal management. 10 In addition to the physician's inexperience, anatomy changes, such as a deviated septum, and materials that have a rougher surface can damage the nasal mucosa. 1 Standardizing the treatment may be beneficial.

The objective of the present study is to describe proper characteristics for an effective nasal foam packing for adult epistaxis, as well as determine pressure supported by the packing, best foam dimensions and density, and its functionality for epistaxis control.

## Methods

**Fig. 1:**
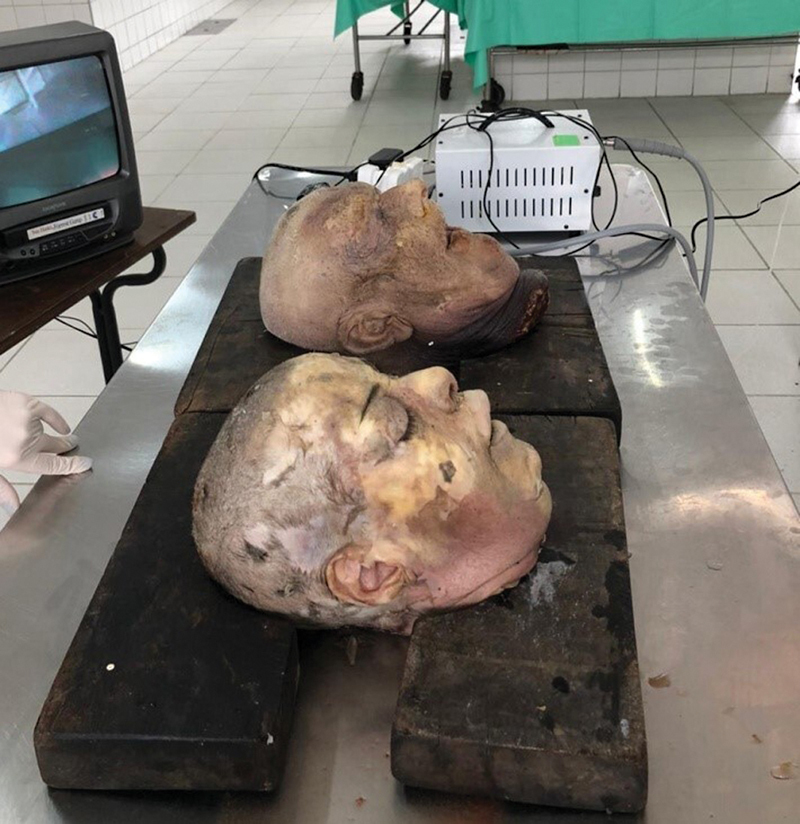
Heads positioned on wooden support. Anterior: female head. Posterior: male head.

The present study is an experimental analytical type, submitted to the Ethics Committee of Universidade de Taubaté (UNITAU), under the protocol number 36030520.8.0000.5501, carried out in the university's Laboratory of Anatomy, on two heads of corpses of unknown individuals, one male, and one female, preserved by the freezing method. The nasal pyramids measured 10 cm in the female head and 13 cm in the male head, and both had a 24-hour period for the thawing before the beginning of the study, carried out at room temperature ( Fig. 1 ).

The heads were placed in horizontal dorsal decubitus at 30° position using wooden skull support. A perforation of the canine fossa was performed, creating a hole in the lower front-lateral region of the maxillary bone in both face hemispheres. To perform the perforation, a Weitlaner retractor was used to provide a better view of the site to be perforated, followed by a 3 cm horizontal incision in the mucosa with a number 11 scalpel. Only the left side perforation in both heads was used due to a fluid outflow during irrigation. The local dissection was completed using a Freer rugina, which removed all the mucous tissue and periosteal until completely exposing the bone tissue. A 3 mm drill surgical motor was used to create an orifice capable of connecting the external region to the interior of the maxillary sinus, with a 0.9% saline set up to the nasal cavity and mimicking epistaxis. The saline solution in the setup was colored with blue dye and connected with ethyl-cyanoacrylate glue in the orifice created.

**Fig. 2:**
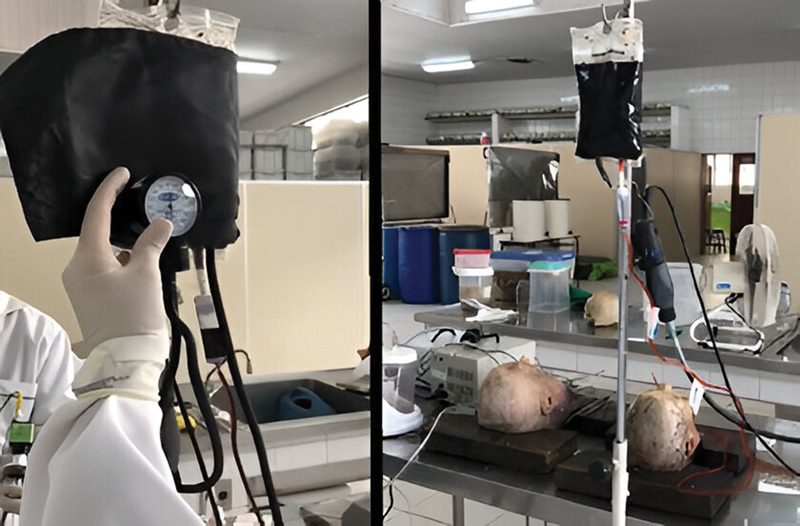
On the left, sphygmomanometer applied under the saline bag, to apply pressure to its outlet. On the right, the equipment connected to the heads for the application of the serum stained in blue.

To identify the maximum pressure supported by the equipment used for nasal packing, a 500 mL bag with the blue-dyed saline was controlled from a Premium© brand cuff with a unit similar to the metered dose in the blood (mmHg). The controlled pressure blue-dyed saline solution entered the nasal cavity through the orifice of the maxillary bone, exiting through the maxillary ostium and descending through the nasal cavity, entering the pharynx, due to head inclination ( Fig. 2 ).

**Fig. 3:**
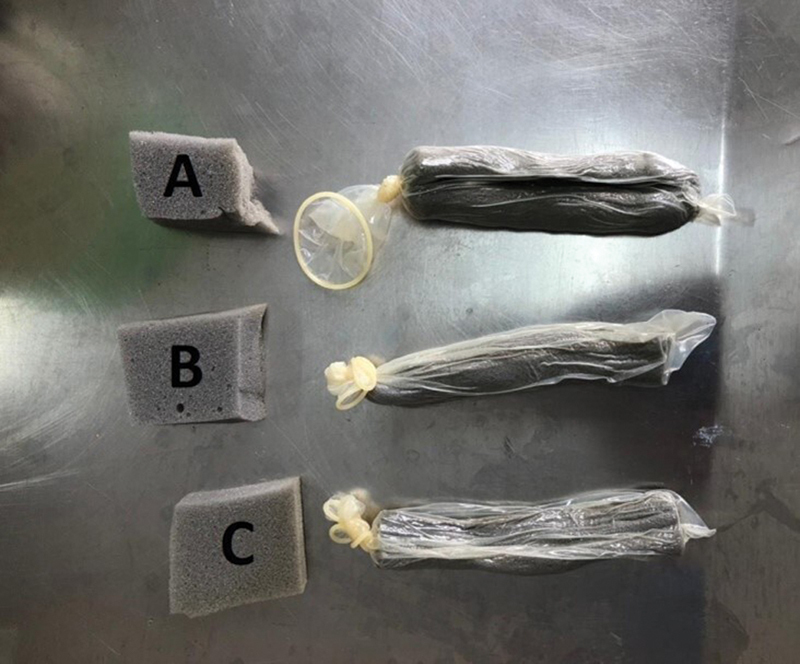
Nasal packings manufactured with foam and condom. A: 1.0cm thick foam d33. B: d33 foam 2.0cm thick. C: foam d33 3.0cmthick.

To control the mimicked bleeding caused by controlled pressure in the cuff, an 11 cm long 33-densityfoam, and 1.0 cm thick was compressed and inserted into a male condom in the nasal cavity, with manual air removal to retract the foam. This process was repeated with other foams, also 11 cm in length and 33 densities, however with different thicknesses, of 2.0 and 3.0 cm ( Fig. 3 ), in addition to a Merocel© foam.

To fit the different tampons and stop the supposed bleeding, a Lucae clamp (bayonet) was used for complete insertion of the packings, which evolved with a small amount of petroleum jelly. The correct packing was verified by visualization through the contralateral nostril, with video equipment consisting of a monitor, light source, fiber optic cable, camera, and a 4 mm endoscope at 30° (Schoelly, Germany).

The pressure was applied by the cuff on the equipment when the packing fit was confirmed. The pressures supported by the plugs were monitored until the liquid came out through the oral cavity. The inlet pressure of the serum was always controlled, passing through variations from 200 mmHg to 280 mmHg to test the ability to contain bleeding by the foams used.

## Results

The results obtained showed a functional superiority of the low-cost material manufactured packings (condoms and foam) in relation to the commercial foam, except for the thinner foam manufactured packing. The pressures supported to contain the mimicking bleeding were higher in foams with thicknesses of 2.0 cm and 3.0 cm ( Table 1 ).

**Table 1:** Maximum pressure supported by each nasal packing

PACKING Density - thickness x length	MAXIMUM SUPPORTED PRESSURE
d33 foam - 1.0cm x 11cm	180mmHg
d33 foam - 2.0cm x 11cm	250mmHg
d33 foam - 3.0cm x 11cm	280mmHg
Merocel® commercial foam	220mmHg

## Discussion

Epistaxis is considered the main otorhinolaryngological emergency. Although it is often stopped spontaneously, approximately 5 to 6% of the cases need fast medical intervention to stop the bleeding. 1 2 Therefore, the physician must take emergency action even without knowing the place of origin or bleeding cause, generating some discomfort for both patient and physician. 2 Thus, easy and fast resolving methods such as ready-made or semi-ready nasal packings are used. In general, the packing effectiveness is lower when compared to the cauterization of a bleeding vessel. However, it is still capable of exerting uniform pressure on the mucosa, enabling the generated edema and the inflammatory process to contain the bleeding. 11 The use of commercial tampons leads to some debating. Commercial nasal packings, such as Merocel©, may cause traumatic damage in placement as it is rigid and rougher, and it is costly. The hospital cost associated with the use of Merocel© is higher than other forms of non-surgical treatment; up to U$50.00. The cost factor is crucial in the Brazilian health system, in which such material is rarely found. Moreover, the fact that the product requires removal and monitoring should be considered. 12 Although the use of foam packing also needs monitoring, the cost of the material would be significantly lower, around U$1. Besides its usage may be less traumatic as the material is malleable and smooth. Very similar good results may be achieved at a much lower cost.

Thus, this project aimed at observing whether the manufactured nasal packing is easy to place, less harmful and whether it tolerates high pressure to control bleeding. It was found during the procedures that the foam and condom packing tolerates high pressure, reaching 280 mmHg, while Merocel® packing, a dehydrated compressed sponge composed of hydroxylated polyvinyl acetate, 13 controlled bleeding at a lower applied pressure (220mmHg). According to some studies, Merocel© presents a success rate of 91.5% 13 and 96%. 14 In other studies, it was observed that this packing presented a failure of 23%, and 26%, requiring additional intervention for epistaxis at the time of the packing removal. Concerning the packing used in the present study, assembled with foam and condom, one study found no major flaws in the method, which was used in 98% of patients. 11 However, it found a recurrence of 37% of the patients, mainly in the first week after discharge. Fifty percent of the recurrences presented Systemic Arterial Hypertension. 12 Therefore, these alert for continuous control of blood pressure even using nasal packings that demonstrate success when on site. Our study points to the ability of foam and condom packing to withstand greater pressure, thus maybe justifying the lower success with the Merocel© packing.

During the procedure practical performance of the foam and condom packing on cadaveric heads, great difficulty in applying the developed packing was observed due to unusual circumstances, such as a deviated septum, that impairs linear placement, possibly generating more traumatic damage. In addition, it can be observed that the anatomical circumstances, both in relation to the maxillary sinus and nasal cavity depth were an issue to be considered in the manufactured packing, since the cavities presented different sizes in the analyzed heads, in addition to the different depth ranges by the foams. The procedure enabled a comparison to the application in patients based on studies, which also demonstrated a low cost and an easy packing performance when compared to surgical methods. Among the patients' sample, 14.8% presented septal deviation, 14.8% concha hypertrophy, and 11.1% these associated alterations. 15 This former study concluded that there was discomfort in applying the packing, associated with some complications, such as dysphagia, hypoxia, tubal dysfunction, apnea, among others, however with no failure in treatment.

In the present study, it was possible to observe the difficulty in the packing assembling, which took about 4 minutes, as all the air in the foam, which had to be removed manually with the placement of the condom, and a maximum vacuum had to be maintained to facilitate the foam introduction. However, this problem can be solved with a pre-assembly of the packaging, keeping it sterilized and ready for use.

Although these difficulties mentioned above, it could be observed that the nasal packings created from lower-cost materials (foam and condom) brought a superior result in containing the artificial bleeding created by the simulation, especially concerning the commercial tampon used as a comparison since the manufactured packing was able to stop the mimicking bleeding at a higher pressure.

Concerning the foam thickness, it was observed that the thicker the foam is, the more difficult the placement will be. Moreover, compared to the thickness difference from 2.0 to 3.0 cm, the pressure increased to 280 mmHg in the thicker one, which is an uncommon pressure, thus suggesting a 2.0 cm thick foam use.

The lack of a real parameter for the packing application into the nasal cavity prevented us from recognizing the challenges of using the manufactured foam nasal packing daily: the speed of management, dexterity, and, above all, the inconvenience of applying it to a patient for epistaxis.

## Conclusion

The most suitable foam for the condom packing was 2.0 cm thick, 11.0 cm long, and density 33 one, withstanding a commonly encountered pressure of up to 250 mmHg. Its lesser thickness provides a less difficult placement.

In cadavers, the foam packing withstands pressure up to 30 mmHg above the Merocel© one.
